# Collaborative Filtering Recommendation on Users’ Interest Sequences

**DOI:** 10.1371/journal.pone.0155739

**Published:** 2016-05-19

**Authors:** Weijie Cheng, Guisheng Yin, Yuxin Dong, Hongbin Dong, Wansong Zhang

**Affiliations:** College of Computer Science and Technology, Harbin Engineering University, Harbin, Heilongjiang, China; Beihang University, CHINA

## Abstract

As an important factor for improving recommendations, time information has been introduced to model users’ dynamic preferences in many papers. However, the sequence of users’ behaviour is rarely studied in recommender systems. Due to the users’ unique behavior evolution patterns and personalized interest transitions among items, users’ similarity in sequential dimension should be introduced to further distinguish users’ preferences and interests. In this paper, we propose a new collaborative filtering recommendation method based on users’ interest sequences (IS) that rank users’ ratings or other online behaviors according to the timestamps when they occurred. This method extracts the semantics hidden in the interest sequences by the length of users’ longest common sub-IS (LCSIS) and the count of users’ total common sub-IS (ACSIS). Then, these semantics are utilized to obtain users’ IS-based similarities and, further, to refine the similarities acquired from traditional collaborative filtering approaches. With these updated similarities, transition characteristics and dynamic evolution patterns of users’ preferences are considered. Our new proposed method was compared with state-of-the-art time-aware collaborative filtering algorithms on datasets MovieLens, Flixster and Ciao. The experimental results validate that the proposed recommendation method is effective and outperforms several existing algorithms in the accuracy of rating prediction.

## Introduction

The existing recommendation systems have adopted various methods to derive people’s preferences and interests. All such methods can be divided into three categories: content-based recommendation, collaborative filtering-based recommendation (CF) and hybrid recommendation. Among these three approaches, the collaborative filtering approach is one of the most successful. It requires only users’ past behavior, such as their item ratings, browsing history and purchased items, without requiring more extensive knowledge. Over the past decade, neighbor-based CF and latent factor model-based CF approaches have been proposed, and their effectiveness and efficiency have been verified in recommendation systems. In order to improve the CF approaches, many efforts have been carried out. Zike Zhang et al. utilized social tags to solve well-known cold-start problem in social tagging systems [1]. A scaling-based algorithm with tunable parameters was introduced to promote personalized recommendation in solving the accuracy-diversity dilemma, presenting a high novelty and solving cold-start problem [2]. Based on classical Matrix Factorization (MF), multiple possible pairwise relationship affecting the final rating decisions of online users was extracted and then linearly integrated to predict their ratings [3]. In recent years, time information has been introduced into the CF approaches to model people’s dynamic evolving preferences and interests. Although contradictory conclusions can be drawn from the existing literature (some propose that the weight of more recent items should be increased, while others suggest that older items should not be underweighted), many researchers agree to some extent that users’ preference and interests change constantly over time and that time information indeed influences the performance of recommendation systems. In particular, the championship team from the Netflix Prize competition claimed that time information is crucial to their method [4][5].

However, the existing time-aware collaborative filtering approaches considered the time factor to be only an adaptive weight for adjusting users’ similarities [5][6][7] or rating history [8] directly; they ignored the sequence of the rated items. In our research we find that the sequence is non-trivial for improving recommendation accuracy because correlations and inter-item dependencies can cause various transition probabilities [9] among items for diverse users, and the differences between users’ characteristics and experiences can also lead to users’ unique rating sequences. In addition, sequences can reflect the evolutionary patterns of users’ preferences and experiences, which can to some extent reveal hidden information affecting their experiences and tastes. As every item has various features or functions that meet users’ tastes, the ratings on common items cannot expose the differences in users’ preference transition sequences, but the sequence of their ratings can. Furthermore, inspired from the analysis of temporal spatial sequences in location-based recommendation systems [10][11][12][13][14][15], this paper assumes that the interest sequences (IS) might also carry more semantics than single-interest points in on-line recommendation systems, and these semantics could be used to analyze the evolutionary patterns of users’ real dynamic interests. The assumption holds because users’ interests are not static but dynamic. These dynamic interest processes can be expressed using IS that contain interest transition sequences among items and the sequence of users’ behaviors. To analyze users’ dynamic interests, this paper gives a formal definition of “interest sequences” and designs methods to measure users’ similarities based on the longest common sub-IS (LCSIS) and all common sub-IS (ACSIS). A new similarity measurement method is designed by combining IS-based similarities with classic similarities from traditional CF methods. According to these new similarities, the other users in the system are ranked and then the top *K* users are selected as the target user’s neighbors. The rating prediction method that estimates users’ potential scores on unobserved items is presented using the neighbors’ observed ratings and the similarities among users.

The main contributions of this paper are as follows:

1. To depict users’ dynamic interest evolution patterns, we define the term “interest sequences” and other related concepts in on-line recommendation systems, which are inspired by work in location-based recommendation systems.
2. Based on the interest sequences, we introduce methods to calculate the length of LCSIS and the count of ACSIS, which are extended by taking into account the sequence and the deviations of users’ ratings on the common items.
3. Given the length of LCSIS and the count of ACSIS, we design the method to calculate users’ similarities based on IS, which are used to find the target user’s top *K* nearest neighbors. Finally, we present the method to predict users’ ratings for unrated items.
4. We adopt three datasets (MovieLens [16], Flixster [17] and Ciao [18]) to verify the effectiveness of the proposed recommendation method through comprehensive experiments and discuss the influence of several factors on the results.

## Related Work

This paper proposes a new CF recommendation method based on users’ IS. In particular, we employ the sequential semantics contained in the interest sequences to select the most similar neighbors to predict a user’ preferences to items, so the proper items can be recommended. In this section, we review the most related research from the following two areas: CF recommendation approach and similarity measurement of time series data.

### CF Recommendation Approach

The CF recommendation approach is one of the most successful and widely used recommendation techniques. CF makes predictions about a user’s ratings by collecting interests from many similar users [19]. The philosophy behind CF relies on the assumption that users who have had similar interests in the past are likely to share common interests in the future [20]. For decades, CF has been developed in two main categories: neighbor-based CF and latent factor model-based CF. Although recently, latent factor model-based CF using matrix factorization has obtained some of the best results [21][22][23][24], neighbor-based CF is still quite attractive due to its easy implementation. Neighbor-based CF relies on the similarities among users to select the most similar users as the target user’s neighbors and then predicts the ratings on the unobserved items using the neighbors’ observed ratings. For neighbor-based CF, similarity measurement is a critical design decision, and several different similarity functions have been proposed and evaluated in the literature. The most commonly used similarity functions include the Pearson Correlation Coefficient, Vector Space Similarity and commonly rated items between users [25]. The conventional neighbor-based CF has the great advantage of being able to integrate rich side information from users and items to refine similarities [26]. The most commonly used side information includes timestamps, which record the precise moments at which users interact with an item. As important context information, timestamps can be used to model users’ dynamic interests [27][28] and to tackle the evolution of user preferences [5][8][29][30][31]. Time is usually introduced as an adjusting factor to increase or reduce the weight of a user’s most recent ratings, but the sequence of users’ rating behaviors has rarely been studied; therefore, that topic is the main focus of this paper.

### Similarity Measurement of Time Series Data

Time series data abounds in real world problems [32], and the measurement of their similarity is crucial in various applications such as bioinformatics, web mining and text mining [33]. Due to the sequential and temporal characteristics of time series data, the lengths of the longest common subsequences (LCS) are most commonly used to measure the distance between two time series [33][34][35][36]. CF can use the longest common subsequence as an indication of the similarity relationship between sequences, but that measurement fails to consider common information in other shorter common subsequences [32]. Hui Wang [32] suggested that the common information contained in all the common subsequences (ACS) should also be considered. The count of all common subsequences was introduced to improve the measure of similarity between time series [37][38]. Although the similarity of time series data has been successfully applied in classification problems, it has not been widely used in recommendation systems. Rajhans Mishraa, Pradeep Kumarb and Bharat Bhasker [34] developed a novel system that considered the sequential information present in web navigation patterns to recommend websites to users, and they validated the viability of using LCS-based similarity in recommender systems. However, a similarity technique that integrates LCS length and the ACS count has not yet been studied in recommendation systems.

## Problem Statement

The recommendation method in this paper is based on users’ interest sequences, which consider the sequences of users’ behaviors according to the time of their interactions. To elucidate our method, we provide some notations and definitions here to formalize the recommendation tasks addressed in this paper.

The recommendation task can be considered as a utility function indicating the potential interest of an item for a user. Let *Users* = {*u*_1_, …, *u*_*n*_} denote a set of users, *Items* = {*it*_1_, …, *it*_*m*_} denote a set of items, and *Ts* = {*ts*_1_, …, *ts*_*l*_} denote all the timestamps at which the users rate items. To describe the IS-based recommendation system, we first provide some definitions below.

**Definition 1** An interest point IP stands for a user’s rating on an item at a timestamp, which consists of the user, the item, the rating, and the timestamp, that is, $IP=\{ip_{u,it_{i}}^{ts}=\left(u,it_{i},r_{it_{i}},ts\right)\}$, where *u* is the user, *it*_*i*_ is the item, and *ts* is the rating given by the user *u* on the item *it*_*i*_ at the timestamp *ts*.

**Definition 2** A interest sequence IS stands for a sequence of a user’s interest points according to their time sequence, that is, $is{}_{u}=\left(ip_{u,it_{1}}^{ts_{1}}\to ip_{u,it_{2}}^{ts_{2}}\to \ldots \to ip_{u,it_{l}}^{ts_{l}}\right)$ where *ts*_1_ < *ts*_2_ < … < *ts*_*l*_. As the time sequence matters more than the concrete timestamps in this paper, IS can be written for short as $is{}_{u}=\left(\left(it_{1}^{u},r_{{}_{1}}^{u}\right)\to \left(it_{{}_{2}}^{u},r_{{}_{2}}^{u}\right)\to \ldots \to \left(it_{{}_{l}}^{u},r_{{}_{l}}^{u}\right)\right)$, where *l* is the index that represents an IP’s position in the time sequence.

Based on the above definitions, we can transform all the users’ rating histories into interest sequences. Different from existing methods that use users’ ratings on the common items, this paper utilizes users’ IS to analyze users’ unique preferences because IS carries more semantics than standalone ratings so that it can not only show people’s dynamic interests but also indicate their evolution patterns. To calculate similarities between users’ IS, we take into account the length of the longest common sub-IS and the count of all common sub-IS, which have been verified as effective in classification problems [33][39][40][41]. To achieve the above tasks, we provide some additional definitions as follows:

**Definition 3** Interest Sequence Match (ISM). Given a rating deviation constraint threshold *θ* and two users’ sub-IS $sis{}_{u}=\left(it_{1}^{u},r_{1}^{u}\right)\to \left(it_{2}^{u},r_{2}^{u}\right)\to \ldots \to \left(it_{j}^{u},r_{j}^{u}\right)$ and $sis{}_{v}=\left(it_{1}^{v},r_{1}^{v}\right)\to \left(it_{2}^{v},r_{2}^{v}\right)\to \ldots \to \left(it_{j}^{v},r_{j}^{v}\right)$ from two interest sequences *is*_*u*_ and *is*_*v*_, respectively, these two sub-IS formulate a *j*-length IS match if and only if they satisfy the following two conditions:

1. $\forall i\in \left[1,j\right],it_{i}^{u}==it_{i}^{v}$;
2. $\forall i\in \left[1,j\right],f_{rdev}\left(r_{i}^{u},r_{i}^{v}\right)\leq \theta$;

In the above definition, $f_{rdev}\left(r_{i}^{u},r_{i}^{v}\right)$ is a function to calculate the deviation between the ratings given by user *u* and user *v* for the same item. It can be defined as in Eq (1)

$$\begin{array}{c} f_{rdev}\left(r_{i}^{u},r_{i}^{v}\right)=\left|\frac{r_{i}^{u}-min\left(r^{u}\right)}{max\left(r^{u}\right)-min\left(r^{u}\right)}-\frac{r_{i}^{v}-min\left(r^{v}\right)}{max\left(r^{v}\right)-min\left(r^{v}\right)}\right| \end{array}$$ (1)

Due to the diversity of different users’ rating scales (some tolerant users may give 100 percent of the full mark, but other, more fastidious users may give only 70 percent to their favorite items), all the users’ ratings should be normalized to the same scale [0, 1]. If their deviation is smaller than the deviation constraint threshold *θ*, the two users’ ratings can be considered equal. A smaller *θ* means a stricter similarity constraint, but a too-strict similarity constraint will limit the effect of IS in the recommendation. Therefore, an appropriate *θ* should be optimized according to an application’s sensitivity to IS.

With the defined ISM, LCSIS and ACSIS can be defined as follows:

**Definition 4** Longest Common Sub-IS (LCSIS). A ISM between two interest sequences is an LCSIS if and only if there is no other longer ISM detected between them.

**Definition 5** All Common Sub-IS (ACSTIS). ACSIS counts all the ISM of two interest sequences, including empty ISM.

LCSIS and ACSIS both provide shared common information between two users’ interest sequences. Intuitively, two users are more similar to each other if they have a longer LCSIS and more ACSIS. The similarity calculation based on LCSIS and ACSIS will be given in the following section. Therefore, the recommendation task addressed in this paper can be defined as the IS-based rating prediction problem, which consists of estimating the utility of items for users using the ratings of their nearest neighbors, who are ranked according to their similarity based on users’ LCSTIS and ACSTIS.

## The New Recommendation Method

### Similarity Based on LCSIS and ACSIS

In this paper, similarities between users are calculated considering users’ interest sequences. As defined in the previous section, IS can be regarded as time series data that consists of pairs of items and their ratings sorted by time sequence as indicated by the index. As an important indication of the similarity relationship between time series data, LCS has become one of the most commonly used indications [32][33][41]. Recently, Hui Wang [32] has verified that ACS also contains some common information to a certain degree, and ACS-based similarity is competitive for classification problems. Therefore, we assume that users who have longer LCSIS and more ACSIS should also have more similarity in their preferences. The effectiveness of similarity based on LCS and ACS in traditional online recommendation systems should be studied. However, the existing LCS and ACS algorithms cannot satisfy the need to discover the length of LCSIS and the count of ACSIS directly, as they do not incorporate the items and the rating values between two items at the same time in the matching process. Therefore, two extended methods are proposed based on the algorithms proposed in [32][38].

Consider the two IS *is*_*u*_ and *is*_*v*_ extracted from the rating history of users *u* and *v*, respectively, where |*is*_*u*_| = *m* and |*is*_*v*_| = *n*. Let *ω* be an (*m* + 1) × (*n* + 1) matrix. Then, the length of LCSIS between *u*’s IS and *v*’s IS, denoted by |*lcsis*(*u*, *v*)|, can be calculated in Eq (2):

$$\begin{array}{c} \left|lcsis(u,v)\right|=\omega \left[i,j\right]=\left\{\begin{array}{c} 0,\;\;\;\;\;if\;i=0\;or\;j=0\\ \omega [i-1,j-1]+1,\\ \;\;\;\;\;\;if\;it_{x}^{u}=it_{j}^{v},f_{rdev}\left(r_{i}^{u},r_{j}^{v}\right)\leq \theta \\ max(\omega [i,j-1],\omega [i-1,j]),\\ \;\;\;\;\;\;if\;it_{x}^{u}\neq it_{j}^{v}orf_{rdev}\left(r_{i}^{u},r_{j}^{v}\right)>\theta ) \end{array}\right. \end{array}$$ (2)

where 0 ≤ *i* ⩽ *m* and 0 ≤ *j* ⩽ *n*. Then, |*lcsis*(*u*, *v*)| = *ω*[*m*, *n*].

**Example 1**. Consider two interest sequences *is*_*u*_ and *is*_*v*_ in Table 1 with *Items* = {*C*, *D*, *E*, *I*}. For any item *it* ∈ *Items*, let $max\left(r_{it}^{u}\right)=max\left(r_{it}^{v}\right)=5.0$ and $min\left(r_{it}^{u}\right)=min\left(r_{it}^{v}\right)=0.0$. Besides, let *θ* = 0.2. The set of LCSIS of *is*_*u*_ and *is*_*v*_ is {*C* → *I*, *E* → *I*} and thus |*lcsis*| = 2. The calculation of |*lcsis*(*u*, *v*)| can be performed as below:

$$\begin{array}{c} \begin{array}{cc} \left|lcsis(u,v)\right| & =\omega \left[4,4\right]\\ & =\omega \left[3,3\right]+1\\ & =max(\omega \left[3,2\right],\omega \left[2,3\right])+1\\ & =max(max\left(\omega \left[3,1\right],\omega \left[2,2\right]\right),\omega \left[2,3\right])+1\\ & =max(max\left(max\left(\omega \left[3,0\right],\omega \left[2,1\right]\right),\omega \left[2,2\right]\right),\omega \left[2,3\right])+1\\ & =max(max\left(max\left(0,\omega \left[1,0\right]+1\right),\omega \left[2,2\right]\right),\omega \left[2,3\right])+1\\ & =max(max\left(1,\omega \left[2,2\right]\right),\omega \left[2,3\right])+1\\ & =max(max\left(1,max\left(\omega \left[2,1\right],\omega \left[1,2\right]\right)\right),\omega \left[2,3\right])+1\\ & =max(max\left(1,max\left(1,max\left(\omega \left[1,1\right],\omega \left[0,2\right]\right)\right)\right),\omega \left[2,3\right])+1\\ & =max(max\left(1,max\left(1,max\left(\omega \left[1,0\right],\omega \left[0,1\right]\right)\right)\right),\omega \left[2,3\right])+1\\ & =max(1,max\left(\omega \left[2,2\right],\omega \left[1,3\right]\right))+1\\ & =max(1,max\left(1,\omega \left[0,2\right]+1\right))+1\\ & =2 \end{array} \end{array}$$

**Table 1:** Example of two interest sequences.

	*t*_1_	*t*_2_	*t*_3_	*t*_4_
*is*_*u*_	(*C*,2.5)	(*E*,3.0)	(*D*,4.5)	(*I*,0.5)
*is*_*v*_	(*E*,4.0)	(*D*,2.5)	(*C*,3.5)	(*I*,1.5)

The count of ACSIS between *is*_*u*_ and *is*_*v*_, denoted by |*acsis*(*u*, *v*)| (this method is not suitable for interest sequences containing repetitive items, and most recommendation systems do not consider repetitive items in the datasets either) can be calculated in the following equation Eq (3):

$$\begin{array}{c} \left|acsis(u,v)\right|=\omega \left[i,j\right]=\left\{\begin{array}{c} 1,\;\;\;\;\;if\;i=0\;or\;j=0\\ \omega [i-1,j],\\ \;\;\;\;\;\;if\;i,j>0,\xi_{v}\left(j,\left(it_{i}^{u},r_{i}^{u}\right)\right)=0\\ \omega \left[i-1,j\right]+\omega [i-1,\xi_{v}\left(j,\left(it_{i}^{u},r_{i}^{u}\right)\right)-1],\\ \;\;\;\;\;\;if\;i,j>0,\xi_{v}\left(j,\left(it_{i}^{u},r_{i}^{u}\right)\right)>0 \end{array}\right. \end{array}$$ (3)

where 0 ≤ *i* ⩽ *m* and 0 ≤ *j* ⩽ *n*. Then, |*acsis*(*u*, *v*)| = *ω*[*m*, *n*].

Note that, different from the traditional LCS and ACS algorithms, the calculations of |*lcsis*(*u*, *v*)| and |*acsis*(*u*, *v*)| have to take the deviation between two users’ ratings on the matching items into consideration. The function $\xi_{v}\left(j,\left(it_{i}^{u},r_{i}^{u}\right)\right)$ shown as part of formula (3) obtains the position *x*(1 ≤ *x* ≤ *j*) where there exists an IP $\left(it_{x}^{v},r_{x}^{v}\right)$ in *is*_*v*_ such that $it_{x}^{v}=it_{i}^{u}$ and $f_{rdev}\left(r_{i}^{u},r_{x}^{v}\right)\leq \theta$. The function is given by Eq (4):

$$\begin{array}{c} \xi_{v}\left(j,\left(it_{i}^{u},r_{i}^{u}\right)\right)=\left\{\begin{array}{cc} x, & if\;it_{x}^{v}=it_{i}^{u},f_{rdev}\left(r_{i}^{u},r_{x}^{v}\right)\leq \theta \\ 0, & otherwise \end{array}\right. \end{array}$$ (4)

where 1 ≤ *x* ≤ *j*.

**Example 2**. Consider two interest sequences *is*_*u*_ and *is*_*v*_ in Example 1. The set of ACSIS of *is*_*u*_ and *is*_*v*_ is {*ϕ*, *C*, *E*, *I*, *C* → *I*, *E* → *I*} and then |*acsis*(*u*, *v*)| = 6. The calculation of |*acsis*(*u*, *v*)| can be performed as below:

$$\begin{array}{c} \begin{array}{cc} \left|acsis(u,v)\right| & =\omega \left[4,4\right]\\ & =\omega \left[3,4\right]+\omega \left[3,4-1\right]\\ & =\omega \left[2,4\right]+\omega \left[3,3\right]\\ & =\omega \left[1,4\right]+\omega \left[1,1-1\right]+\omega \left[3,3\right]\\ & =\omega \left[0,4\right]+\omega \left[0,3-1\right]+1+\omega \left[3,3\right]\\ & =1+1+1+\omega \left[2,3\right]\\ & =3+\omega \left[1,3\right]+\omega \left[1,1-1\right]\\ & =3+\omega \left[0,3\right]+\omega \left[0,3-1\right]+1\\ & =4+1+1\\ & =6 \end{array} \end{array}$$

To compare the similarities between two users’ IS, the normalization of |*lcsis*(*u*, *v*)| and |*acsis*(*u*, *v*)| is conducted in Eqs (5) and (6) as follows:

$$\begin{array}{c} sim{}_{LCSIS}\left(u,v\right)=\frac{\left|lcsis(u,v)\right|}{\sqrt{\left|lcsis(u,u)\right|\times \left|lcsis(v,v)\right|}} \end{array}$$ (5)

$$\begin{array}{c} sim{}_{ACSIS}\left(u,v\right)=\frac{\left|acsis(u,v)\right|}{\sqrt{\left|acsis(u,u)\right|\times \left|acsis(v,v)\right|}} \end{array}$$ (6)

Then, we can use the factor *α* to combine these two types of similarity measurements as defined in Eq (7):

$$\begin{array}{c} sim{}_{IS}\left(u,v\right)=\alpha \times exp^{sim_{LCSIS}\left(u,v\right)}+\left(1-\alpha \right)\times exp^{sim_{ACSIS}\left(u,v\right)} \end{array}$$ (7)

where the factor *α* ∈ [0, 1]. The factors *α* and (1 − *α*) represent the weights of LCSIS and ACSIS in the similarity measurement, respectively. The value of *α* should be set to optimize the recommendation results in different applications.

### An Updated Similarity by Combining IS-based Similarity

We combine our IS-based similarity measure with traditional similarity measures used in existing collaborative filtering recommendation algorithms. We intend to use the user characteristics contained in IS to further improve the recommendation performance. For the Pearson similarity in Eq (8) (for user-based recommender systems, the Pearson similarity outperforms other measures of users’ proximity [42]), the similarity measurement to integrate IS-based similarity is defined in Eq (9):

$$\begin{array}{c} pc\left(u,v\right)=\frac{\sum_{i\in I^{u,v}}\left(r_{i}^{u}-\bar{r^{u}}\right)\left(r_{i}^{v}-\bar{r^{v}}\right)}{\sqrt{\sum_{i\in I^{u,v}}{\left(r_{i}^{u}-\bar{r^{u}}\right)}^{2}}\sqrt{\sum_{i\in I^{u,v}}{\left(r_{i}^{v}-\bar{r^{v}}\right)}^{2}}} \end{array}$$ (8)

$$\begin{array}{c} sim\left(u,v\right)=pc\left(u,v\right)\times f\left(sim{}_{IS}\left(u,v\right)\right) \end{array}$$ (9)

where *pc*(*u*, *v*) is the method to calculate Pearson similarity, and *f*(*sim*_*IS*_) is a weighting function used to reflect the influence of users’ IS on users’ similarity, as given in Eq (10):

$$\begin{array}{c} f\left(sim_{IS}\right)=\frac{comm\left(u,v\right)\times sim_{IS}\left(u,v\right)}{total(u,v)} \end{array}$$ (10)

where *comm*(*u*, *v*) represents the number of common items that user *u* and user *v* have both rated and *total*(*u*, *v*) represents the total of all items that *u* and *v* have rated. The similarity based on IS is *sim*_*IS*_(*u*, *v*), as defined in the previous section.

### The Prediction of Users’ Rating Based on IS

After calculating users’ similarities, we rank all the other users that have rated the target item according to their similarities with the target user and then select the top *K* users as the target user’s neighbors for the target item. For the target user *u*, let $I_{pred}^{u}$ be the items to which the recommender system needs to give predicted ratings for the target user, and let *N*_*u*,*i*_ be *u*’s neighbors on the target item $i\in I_{pred}^{u}$. The equation to predict the rating is shown below in Eq (11):

$$\begin{array}{c} \tilde{r_{i}^{u}}=\bar{r^{u}}+\frac{\sum_{v\in N_{u,i}}sim\left(u,v\right)\left(r_{i}^{v}-\bar{r^{v}}\right)}{\sum_{v\in Nu,i}sim(u,v)} \end{array}$$ (11)

where $\tilde{r_{i}^{u}}$ is the predicted rating given by the user *u* to the item *i*, *v* is one of the nearest neighbors, $r_{i}^{v}$ is *v*’s past rating for item *i*, and $\bar{r^{u}}$ and $\bar{r^{v}}$, respectively, represent the average of *u*’s and *v*’s past ratings.

## Experimental Results and Analysis

To verify the effectiveness of our proposed method, we conduct comprehensive experiments on the real datasets and compare the results with some recommendation methods regarding their performance on two common evaluation metrics. In addition, the influence of the rating deviation constraint threshold *θ* and the weights *α* of LCSIS and ACSIS in similarity calculations with the top *K* neighbors on the results is discussed.

### Dataset Description

For this paper, we need to choose appropriate datasets which contain rating timestamps. Moreover, the datasets must have been collected over a long enough period to contain sufficient interest sequences. For these reasons, this paper uses the following datasets: MovieLens 100k and latest small, Flixster, and Ciao.

The MovieLens dataset [16] was collected by GroupLens Research from the MovieLens web site. The MovieLens data sets were collected over various periods of time, depending on their size. For our experiments, we used the dataset MovieLens 100K, which contains 100,000 anonymous ratings of approximately 943 movies made by 1682 MovieLens users with rating timestamps spanning from 19 April, 1997 to 22 April, 1998 and MovieLens latest small, which contains 100,023 ratings applied to 8,552 movies by 706 users between April 03, 1996 and January 09, 2016.

The Flixster with timestamps dataset [17] contains 100,000 ratings to 11,011 movies by 2,048 users during the period from November 2005 to November 2009.

The Ciao with timestamps dataset [18] contains 36,065 ratings to 16,861 products by 2,248 users during the period from July 2000 to November 2013.

### Evaluation Metrics

To make the experimental results comparable and reproducible, we adopt two well-known metrics, RMSE (Root Mean Squared Error) and MAE (Mean Absolute Error), to measure the accuracy of the predictions. These two measures (*rmse* and *mae*) can be calculated as shown in Eqs (12) and (13):

$$\begin{array}{c} rmse=\sqrt{\frac{\sum_{u\in Users}\sum_{i\in I_{pred}^{u}}{\left(r_{i}^{u}-p_{i}^{u}\right)}^{2}}{\sum_{u\in Users}\left|I_{pred}^{u}\right|}} \end{array}$$ (12)

$$\begin{array}{c} mae=\frac{\sum_{u\in Users}\sum_{i\in I_{pred}^{u}}\left|r_{i}^{u}-p_{i}^{u}\right|}{\sum_{u\in Users}\left|I_{pred}^{u}\right|} \end{array}$$ (13)

where *u* ∈ *Users* denotes the user, $i\in I_{pred}^{u}$ denotes one of *u*’s unrated items, $r_{i}^{u}$ denotes the real rating given by *u* to *i*, $p_{i}^{u}$ denotes the predicted rating given by *u* to *i*, and $\left|I_{pred}^{u}\right|$ denotes the number of *u*’s unrated items. It is clear that lower *rmse* and *mae* values indicate a better recommendation accuracy.

### Experimental Setup

To evaluate the performance of our method and the effectiveness of IS for recommendations, we compare it with the traditional user-based CF recommendation algorithm and a recommendation algorithm based on users’ dynamic information from [8]:

1. User-based CF (UCF) [43]: is a comparative algorithm that uses the rating history of users to calculate the similarities between them and then makes automatic predictions based on those similarities and neighbors’ ratings.
2. The recommendation based on users’ dynamic information (UDI) [8]: is a comparative algorithm that takes users’ dynamic interests into consideration by introducing a decreasing time function to model users’ dynamic interest features.

In our experiments, we refer to our proposed method as ISCF. The existing dataset is split into a training set (80%) and a test set (20%). To avoid overfitting problems, we conduct 5-fold cross-validation experiments. Our work is implemented based on LibRec [44], which is a GPL-licensed Java library for recommender systems.

### Experimental Comparison for Three Methods on Four Datasets

**Fig 1:**
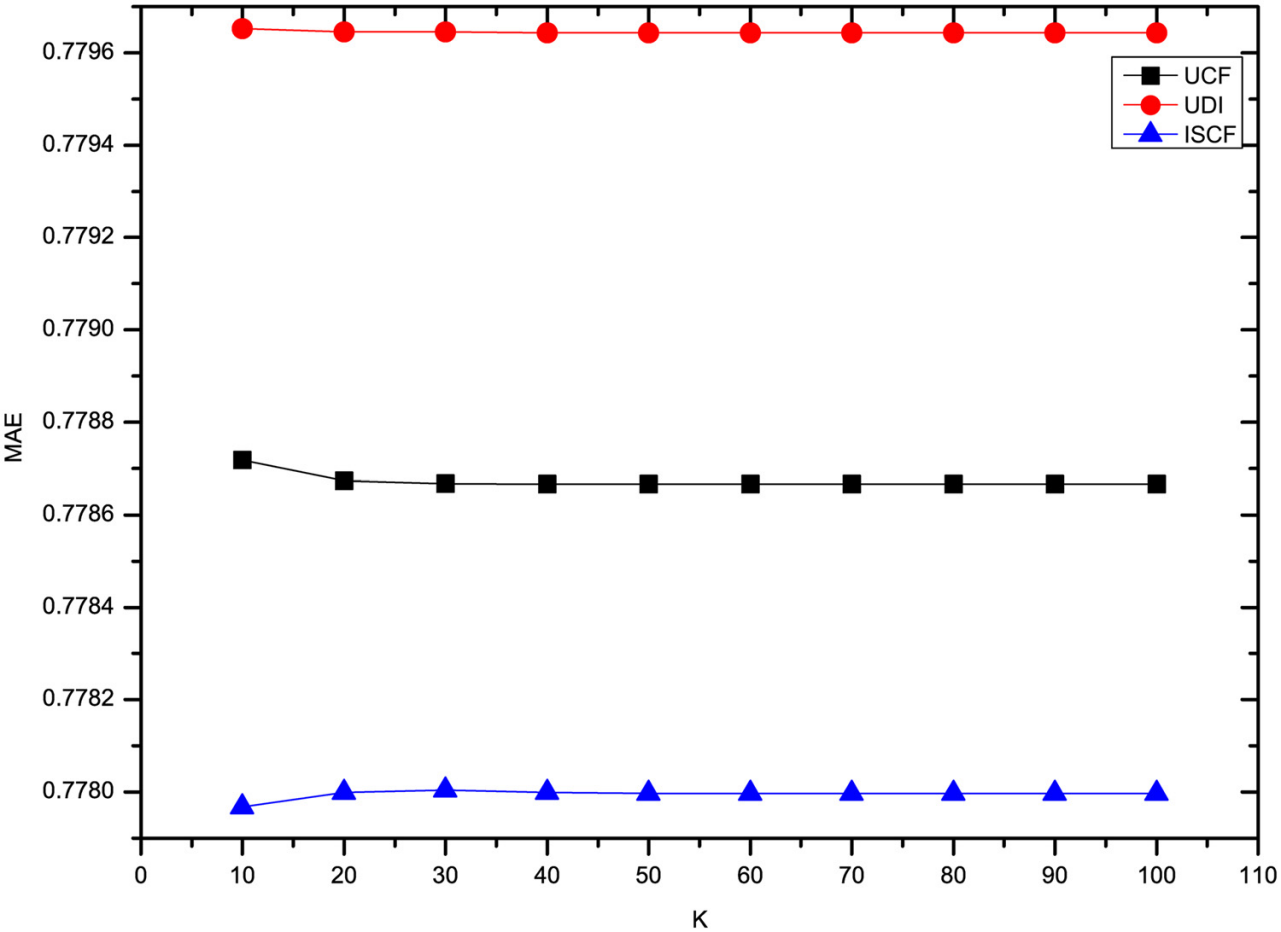
Comparison of three methods’ MAE on Ciao.

**Fig 2:**
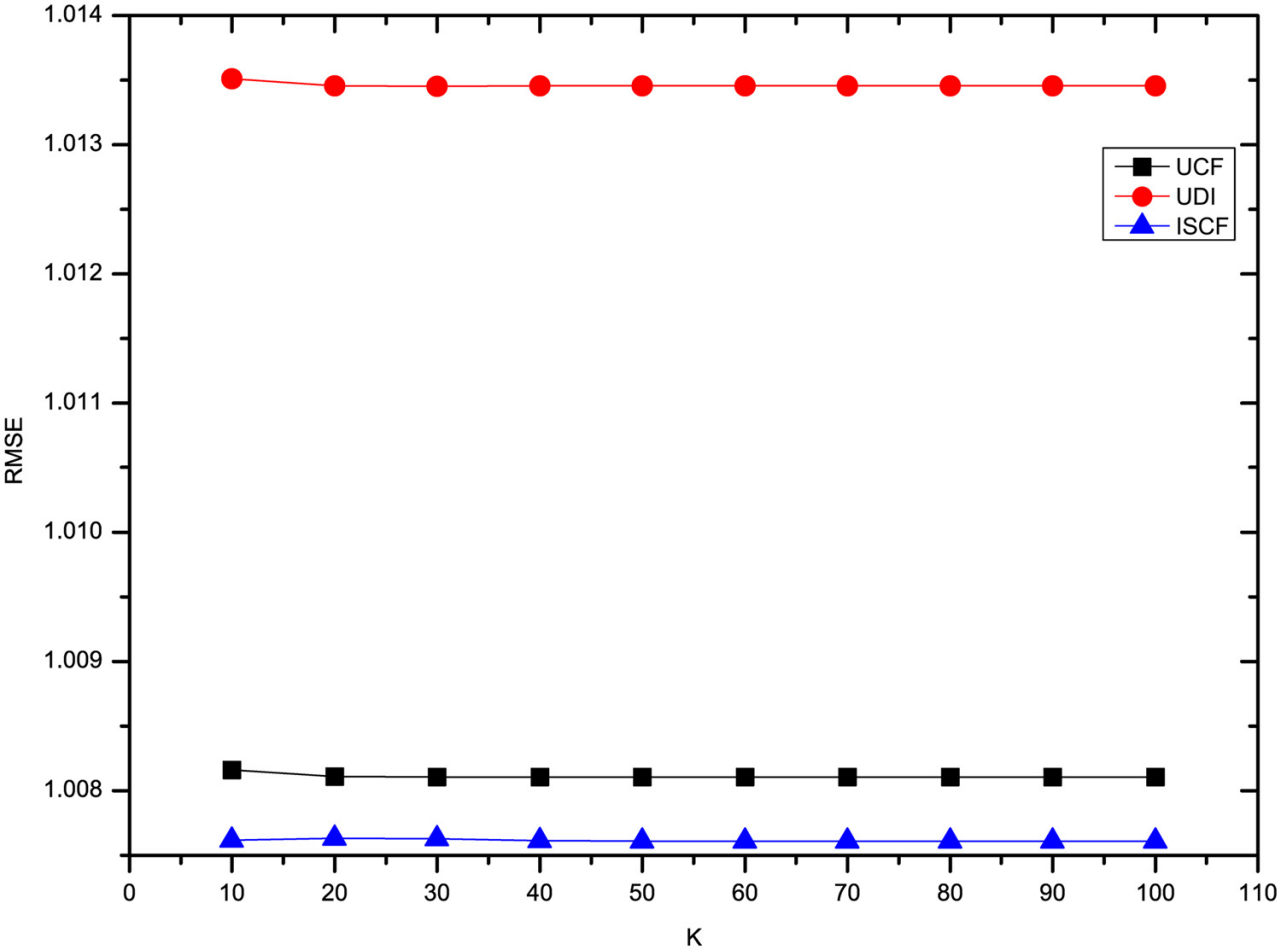
Comparison of three methods’ RMSE on Ciao.

This section compares the results of the experiments using three methods on four datasets. To comprehensively compare the results, the *K* number of neighbors was set to 10, 20, 30, 40, 50, 60, 70, 80, 90 and 100. To compare our results with UDI’s best results, some experiments for optimizing UDI’s results were also conducted. As described in [8], the time decay rate *λ* should be optimized. From the results, we find that UDI performs best in terms of MAE and RMSE when *λ* is 0.1 for four datasets. In addition, for our method ISCF, the rating deviation constraint threshold *θ* was set to 0.8 and the weight *α* of LCSIS in similarity calculation was set to 0.5. Figs 1 and 2 show the results of three methods’ MAE and RMSE on the dataset Ciao. From Figs 1 and 2, it can be seen that three methods perform nearly steadily with increasing numbers of neighbors. ISCF is the best and UDI performs even worse than UCF.

**Fig 3:**
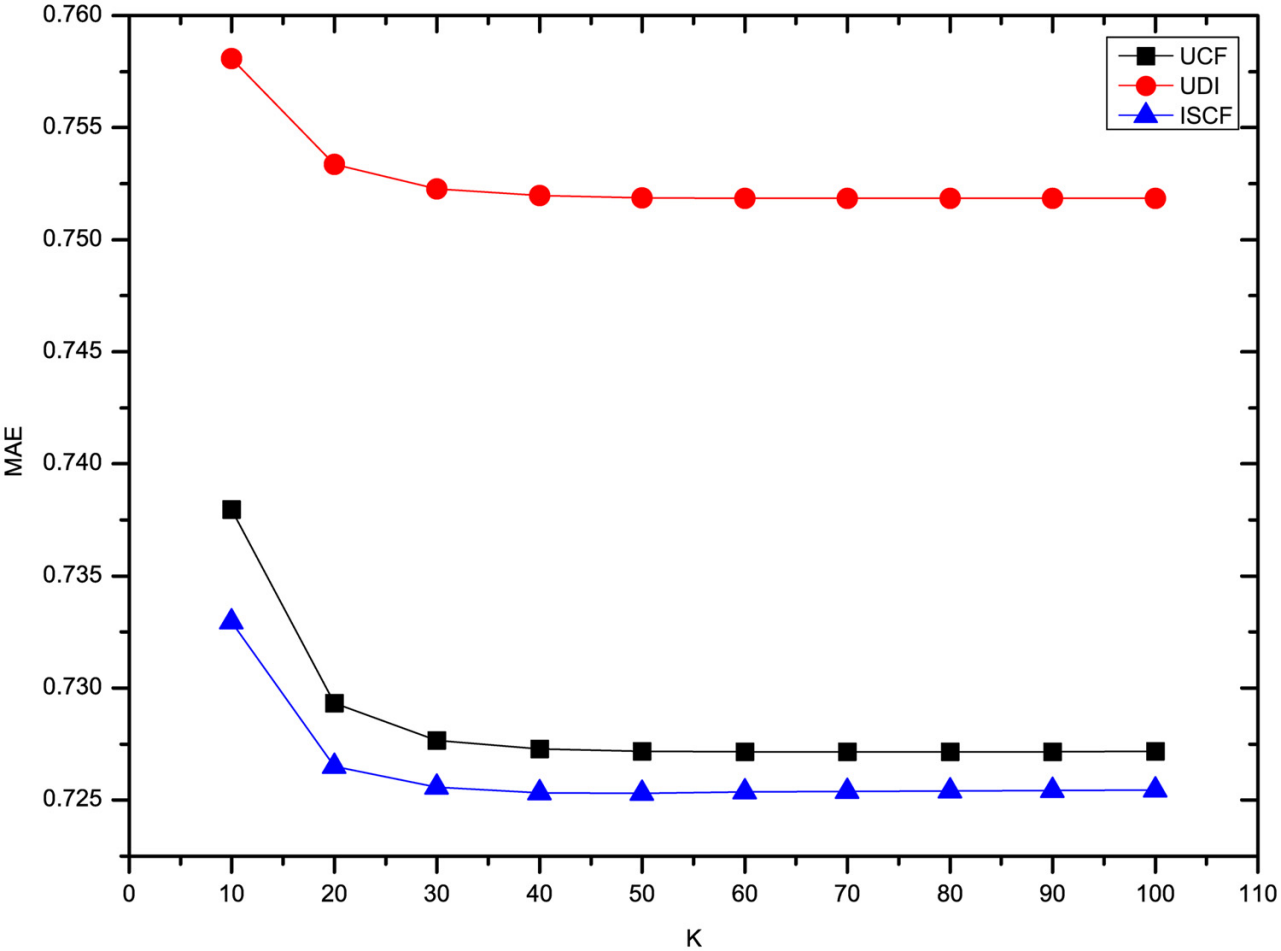
Comparison of three methods’ MAE on Flixster.

**Fig 4:**
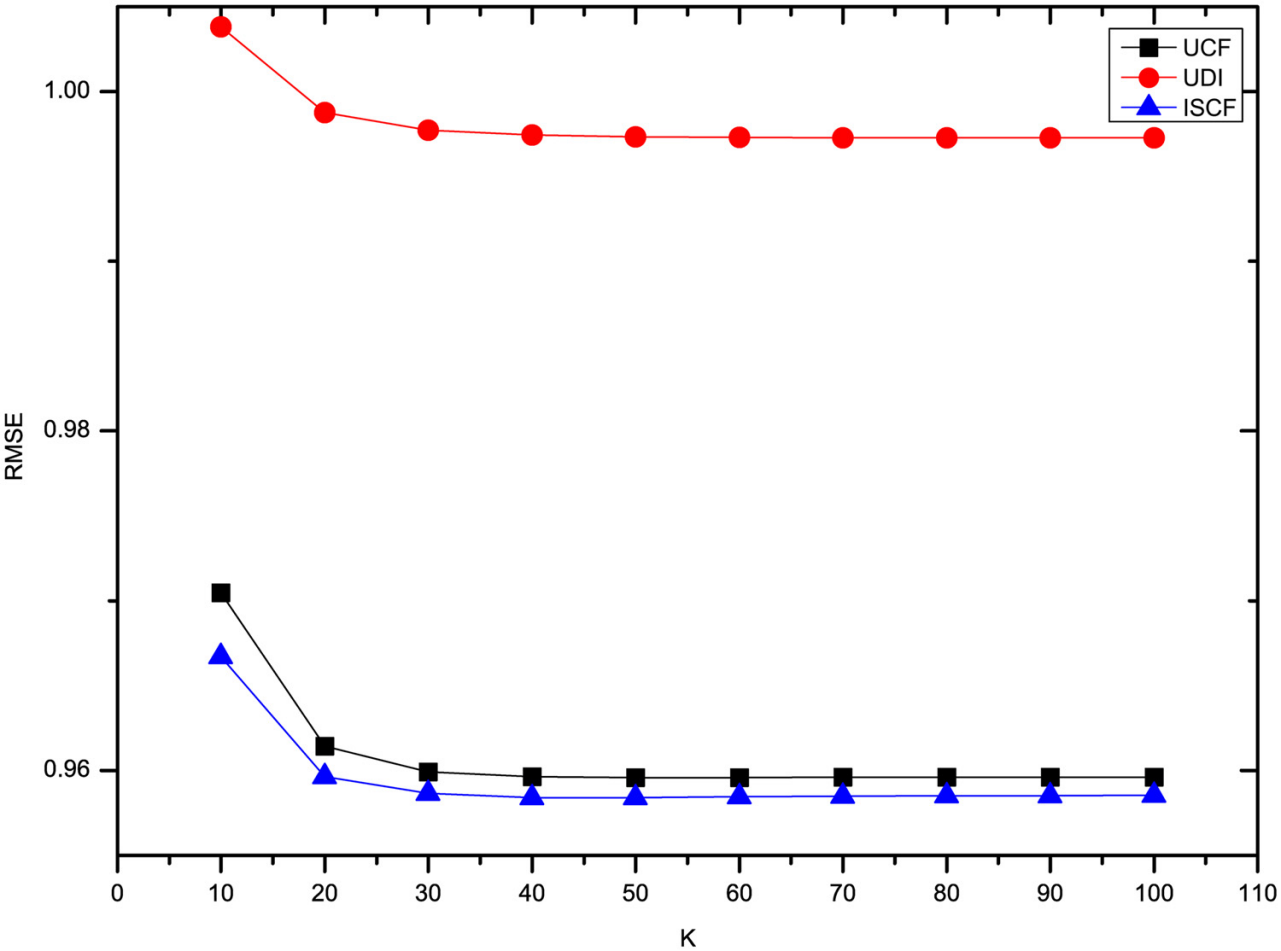
Comparison of three methods’ RMSE on Flixster.

The results of three methods’ MAE and RMSE on the dataset Flixster are shown in Figs 3 and 4. It shows that the precision of rating prediction for three methods is the worst when *K* = 10, and then is improved significantly when 10 more neighbors are introduced. Afterwards, the precision increases slightly and then keeps nearly the same with more neighbors’ ratings utilized. Besides, ISCF has the best performance in the three methods no matter how many neighbors are involved in predicting users’ ratings.

**Fig 5:**
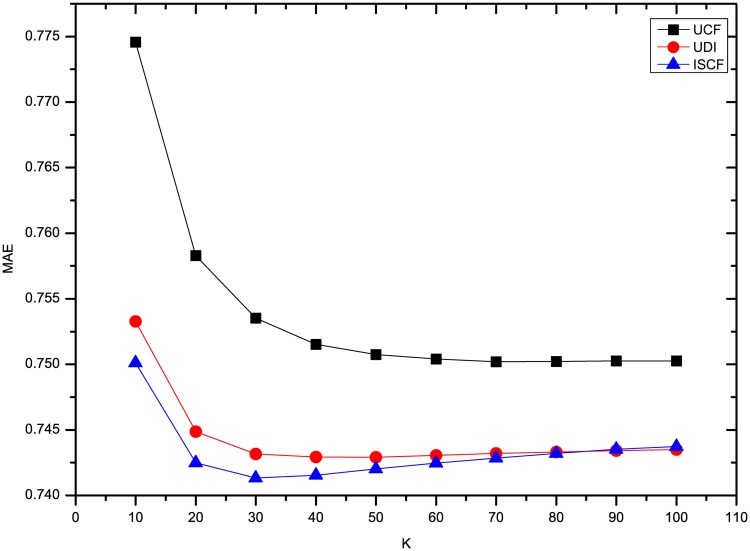
Comparison of three methods’ MAE on MovieLens 100k.

**Fig 6:**
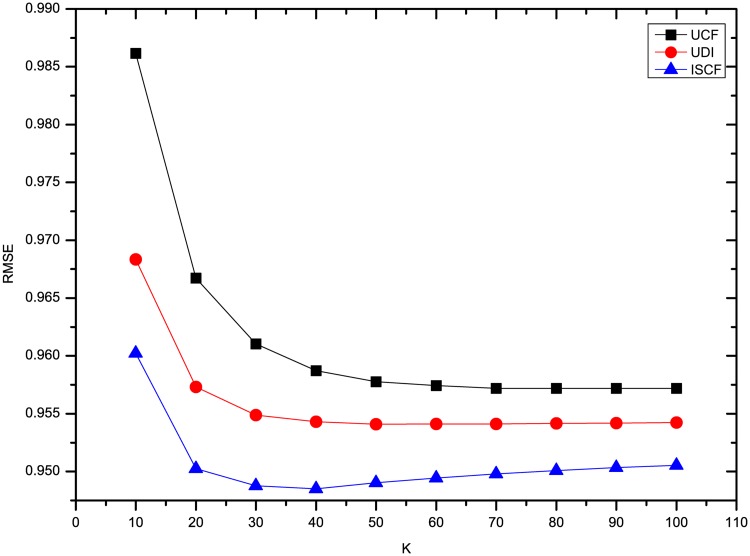
Comparison of three methods’ RMSE on MovieLens 100k.

The comparison of three methods’ MAE and RMSE on the dataset MovieLens 100k is shown in Figs 5 and 6. It demonstrates that ISCF and UDI perform better than UCF, and in Fig 5, UDI’s MAE approaches ISCF’s when the number of neighbors is more than 80. However, UDI still performs not as well as ISCF in RMSE. Based on the results in Figs 5 and 6, it can be verified that ISCF outperforms UCF and UDI on the dataset MovieLens 100k.

**Fig 7:**
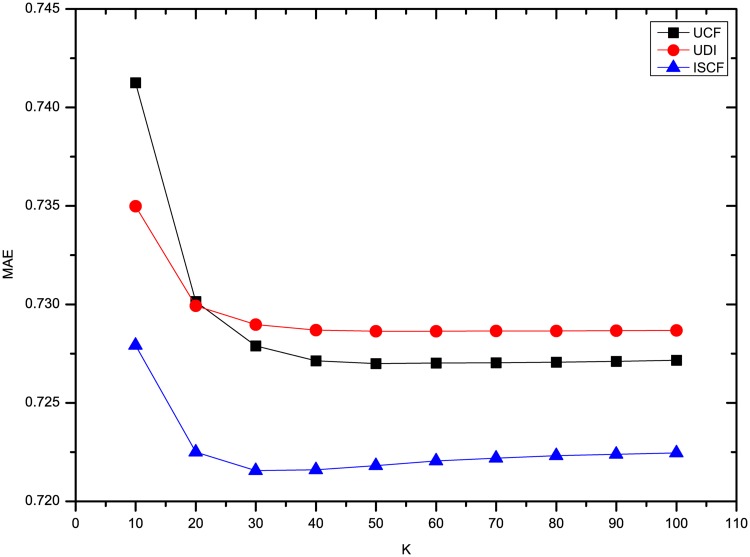
Comparison of three methods’ MAE on MovieLens latest small.

**Fig 8:**
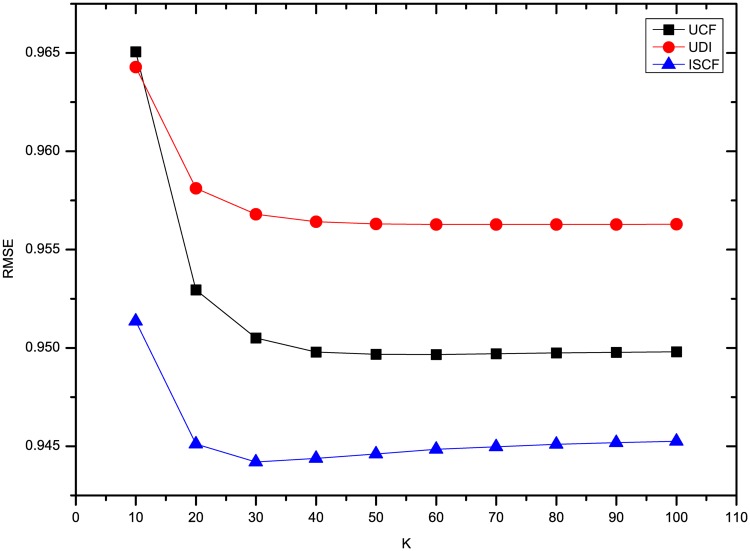
Comparison of three methods’ RMSE on MovieLens latest small.

Figs 7 and 8 give three methods’ results on the dataset MovieLens latest small. It can be seen that UDI performs the worst except the results when *K* = 10 and ISCF has the most precise output.

To summarize, our proposed method ISCF has the best performance in predicting users’ ratings to unknown items in all the four datasets. Although UDI performs better than UCF on the dataset MovieLens 100k, it performs the worst on the other three datasets. Therefore, the effectiveness of interest sequences for improving precision of the recommendation results is verified.

### The Influence of *θ* and *α*

In order to evaluate the influence of LCSIS and ACSIS on the recommendation results, we adopt different values of the rating deviation constraint threshold *θ* and the weight *α* of LCSIS for our proposed method. *θ* was set to 0.2, 0.5 and 0.8, which represent weak, medium and strong constraint for users’ rating deviations, respectively. Meanwhile, *α* was set to 0.2, 0.5 and 0.8 to denote the weight of LCSIS in calculating users’ similarities based on interest sequences. The expected results were supposed to be that different values of *θ* and *α* lead to different recommendation precision. However, the recommendation precision nearly keeps the same. After investigating users’ similarities for different *θ* and *α*, it is found that users’ similarities only have tiny fluctuations (e.g. in the dataset MovieLens latest small, the similarities for different *θ* and *α* between the user 599 and the user 194 in Table 2). The reason is that users’ rating history is of high sparsity and thus the lenghth of LCSIS and the count of ACSIS cannot have significant changes for different *θ* and *α*. Nevertheless, the lenghth of LCSIS and the count of ACSIS are effective in improving the recommendation precision as shown in experimental comparisons for three methods on four datasets.

**Table 2:** Example of two users’ similarities in MovieLens latest small for different *θ* and *α*.

	*α* = 0.2	*α* = 0.5	*α* = 0.8
*θ* = 0.2	0.2270	0.2271	0.2273
*θ* = 0.5	0.2270	0.2271	0.2273
*θ* = 0.8	0.2271	0.2272	0.2273

## Conclusion and Future Work

This paper proposed a new recommendation method based on users’ interest sequences to capitalize on the evolutionary patterns and time sequences of users’ preferences to improve recommendations. First, we formally defined the problem and provided a definition for interest sequences, LCSIS and ACSIS. Then, a similarity measurement method considering LCSIS and ACSIS was proposed. Furthermore, LCSIS and ACSIS-based similarity was combined with traditional similarity to obtain users’ similarity based on users’ interest sequences. In addition, given the IS-based similarity, we select the top *K* most similar neighbors to predict users’ ratings on unknown items. Finally, comprehensive experiments using four datasets validated the effectiveness of IS in improving recommendation accuracy. Moreover, the experimental results also demonstrated that our method outperforms the traditional user-based CF method and time-aware recommendation method.

In future research work, we will continue to study users’ dynamic behavior patterns and analyze characteristics of their IS to improve the accuracy of recommendation systems by taking other contextual information into account. In addition, we plan to develop new methods that consider users’ social relationships and items’ inner connections. Moreover, the calculation efficiency for determining the LCSIS and ACSIS values remains a challenge.

## Supporting Information

S1 File The detailed data of experiment results. (XLS)
